# Supplementary material for: Diatomite derived hierarchical hybrid anode for high performance all-solid-state lithium metal batteries
Source: Nat Commun. 2019 Jun 6;10:2482. doi: 10.1038/s41467-019-10473-w (PMC6554300; doi:10.1038/s41467-019-10473-w)
Supplement: Supplementary file 1 — Supplementary Information [file 41467_2019_10473_MOESM1_ESM.pdf]

Supplementary Information

Diatomite derived hierarchical hybrid anode for high performance all-solid-state lithium metal batteries

Fei Zhou et al.

Supplementary Figures

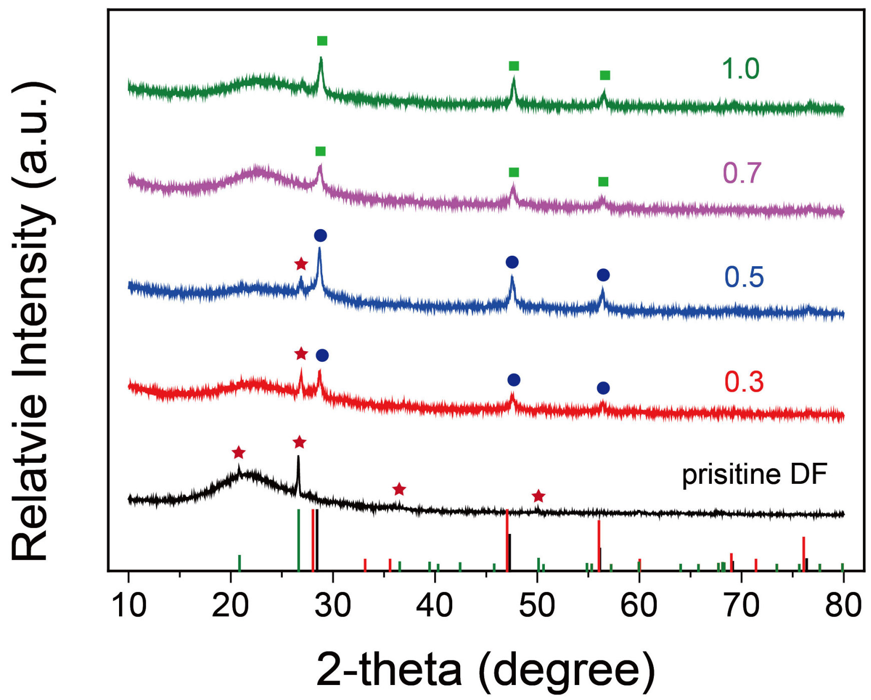

Supplementary Figure 1. PXRD results of diatomite magnesiated with different mass ratio of Mg powder. The perpendicular line in green color is standard peaks of SiO_2 (JCPDS 46-1045), while red color is SiO (JCPDS 30-1127) and black color is Si (JCPDS 27-1402), respectively.

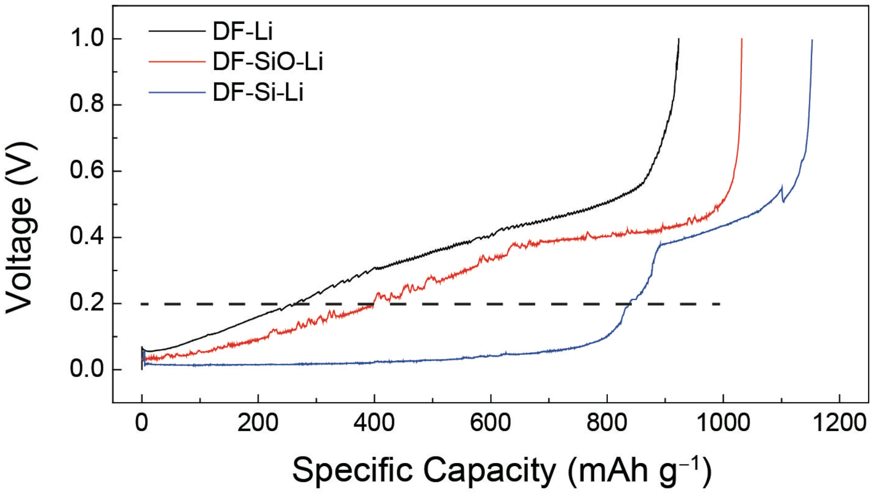

Supplementary Figure 2. Li stripping performance of lithiated diatomite, DF-SiO and DF-Si in liquid electrolyte cells.

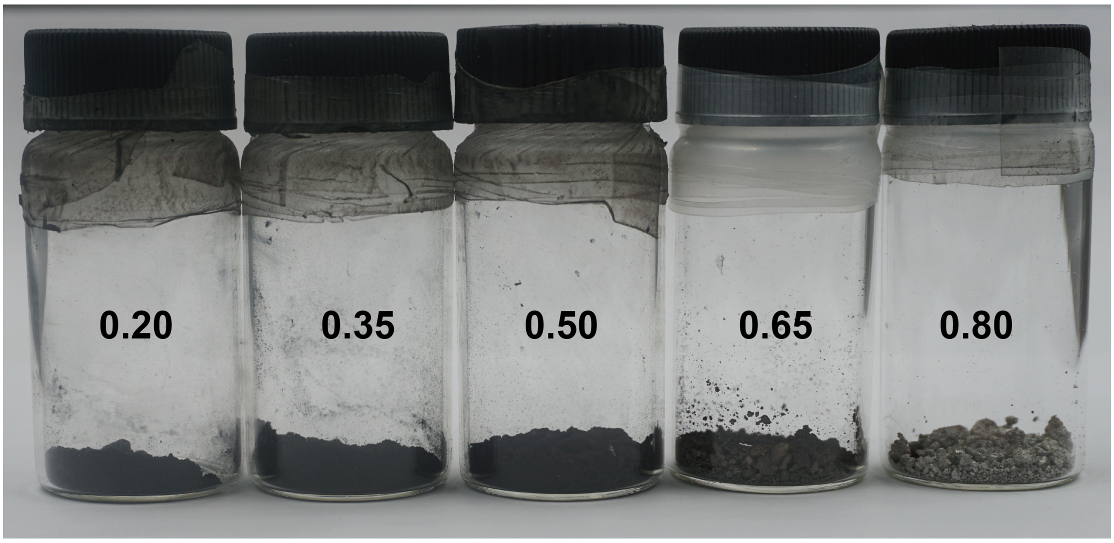

Supplementary Figure 3. Photograph of DF-Si lithiated by different mass of Li. The mass of DF-Si was fixed as 0.5 g.

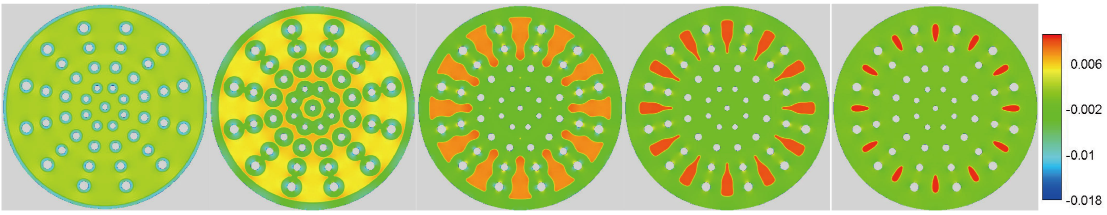

Supplementary Figure 4. Expansion of as-formed lithiated phase. Simulation of DF-Si stress evolution during the lithiation process.

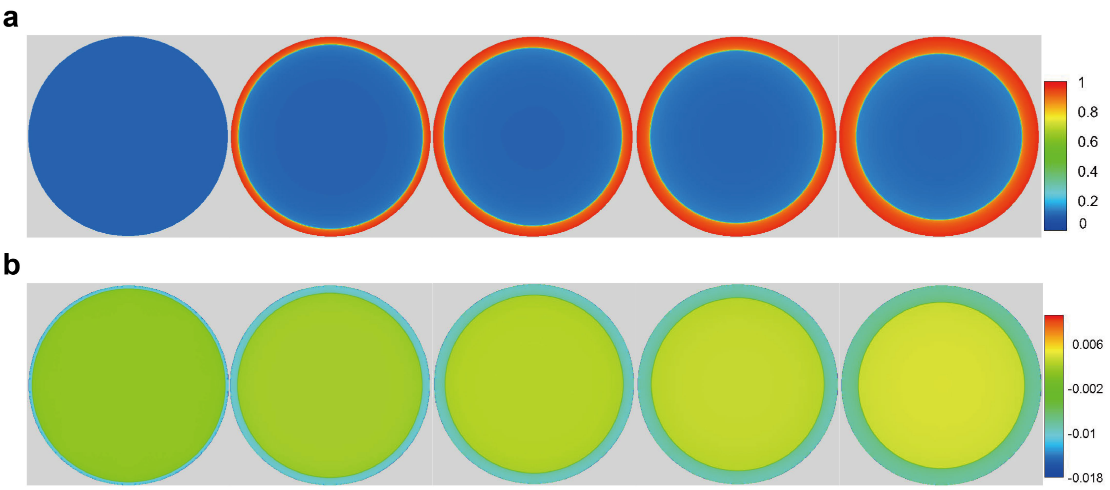

Supplementary Figure 5. Li concentration and stress evolution within the lithiation process. Simulation of (a) Li concentration and (b) stress evolution of Si microflakes during the lithiation process.

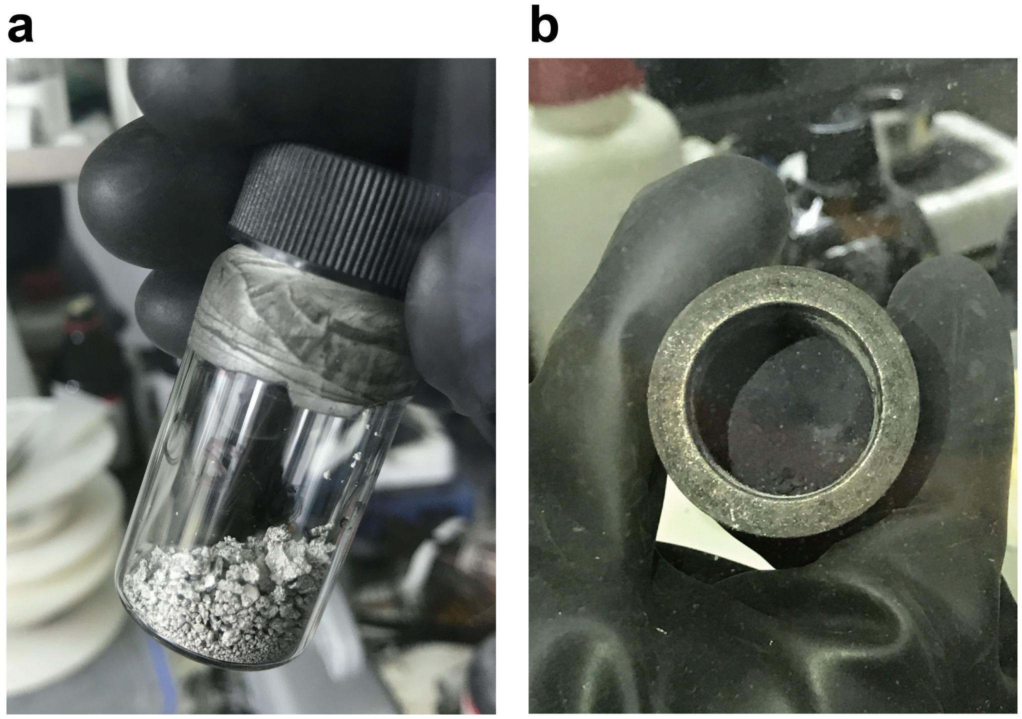

Supplementary Figure 6. Photographs over-stoichiometric lithiation product of DF-Si and Si microparticle powder. (a) DF-Si-$Li_{0.8}$ and (b) Si-$Li_{0.8}$, respectively.

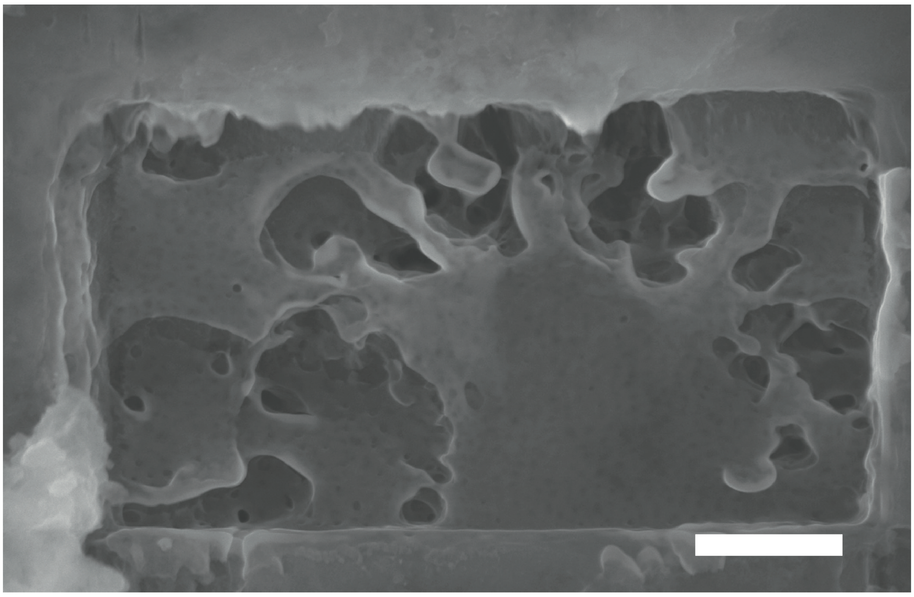

Supplementary Figure 7. SEM image of DF-Si-Li microparticles after focused ion beam etching (Scale bar = 1 μm).

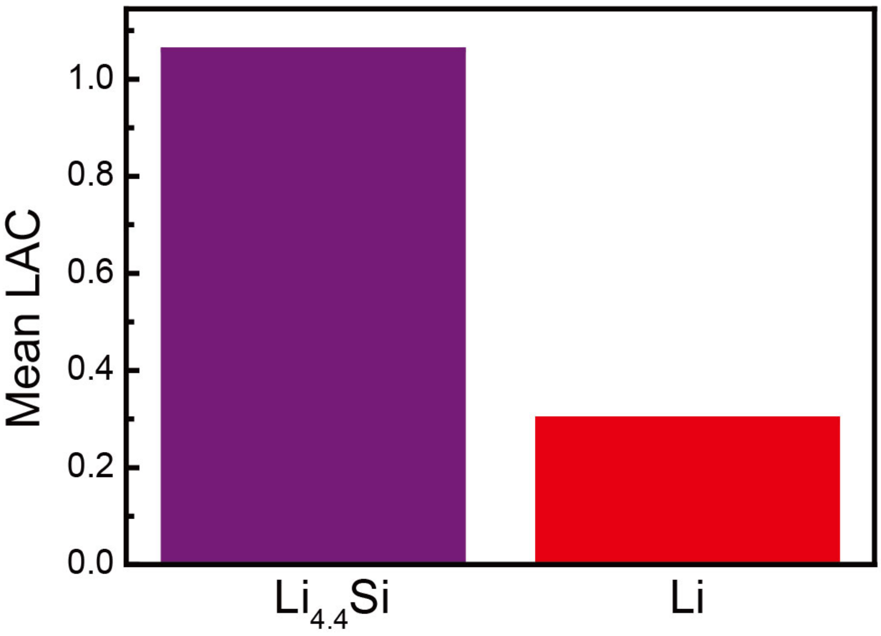

Supplementary Figure 8. The X-ray absorbability (Mean linear absorption coefficient) of $Li_{4.4}Si$ and Li.

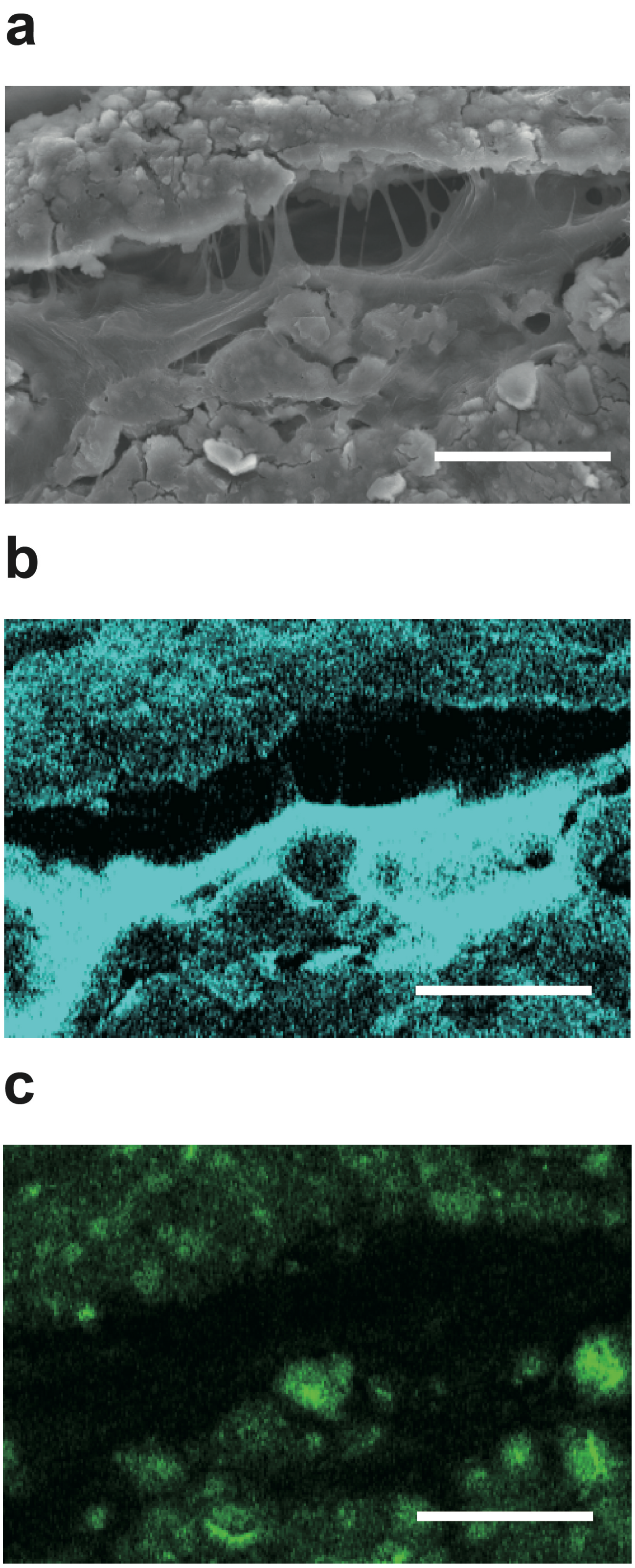

Supplementary Figure 9. SEM imaging and elemental mapping of PEO-DLSL. a, SEM image of the PEO-DLSL electrode at a stretched point. b-c, Corresponding EDX mappings results of the aforementioned area. The blue color represents the signal of oxygen from the PEO-SPE coating, while the green represents the silicon (Scale bar = 25 μm).

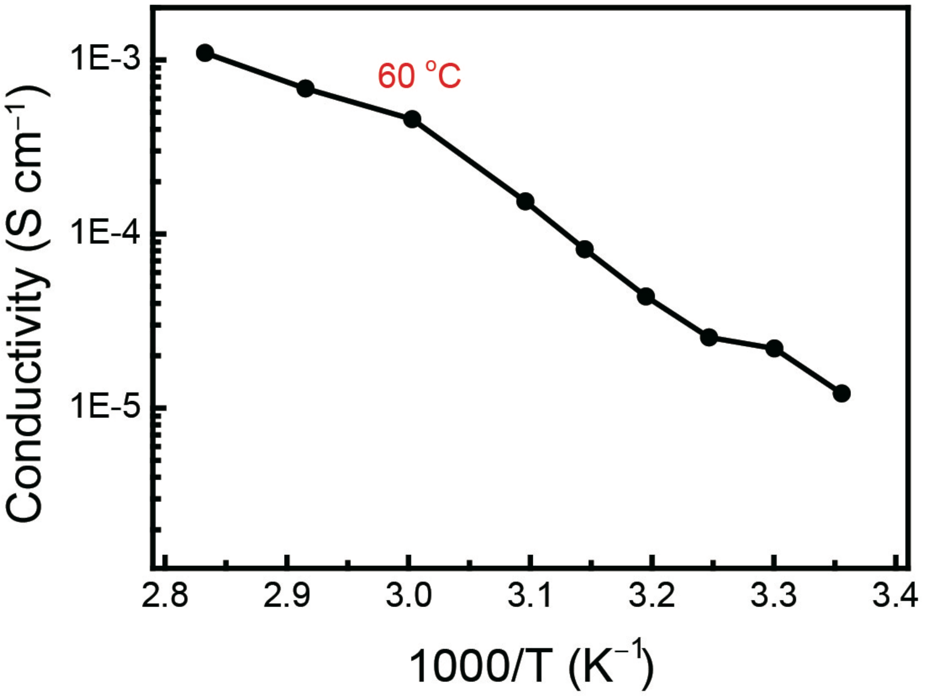

Supplementary Figure 10. Li^+ ion conductivity curve of PEO-PSE at different operating temperature.

a b

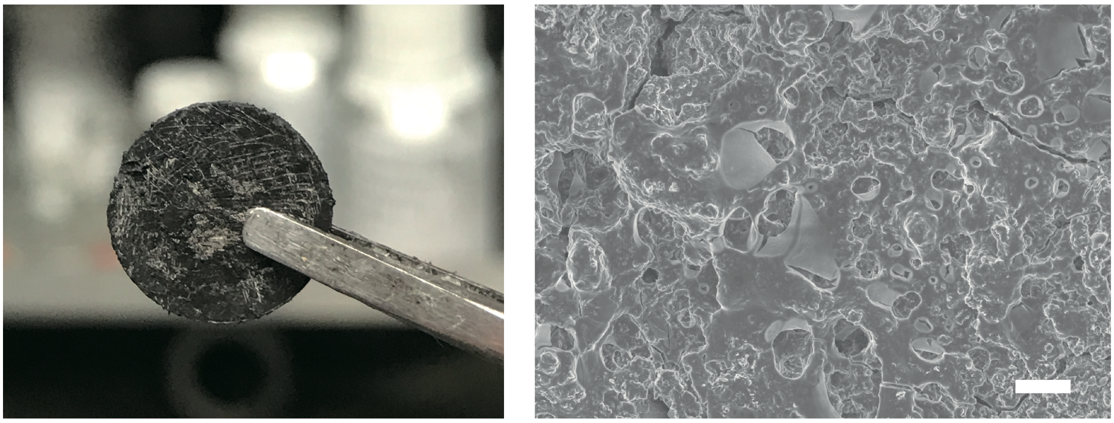

Supplementary Figure 11. Morphology characterization of the delithiated PEO-DLSL. a, Photograph of PEO-DLSL after stripping to 1.0 V vs. Li^+/Li. b, SEM image of the black surface of the PEO-DLSL after the Li stripping (Scale bar = 10 μm).

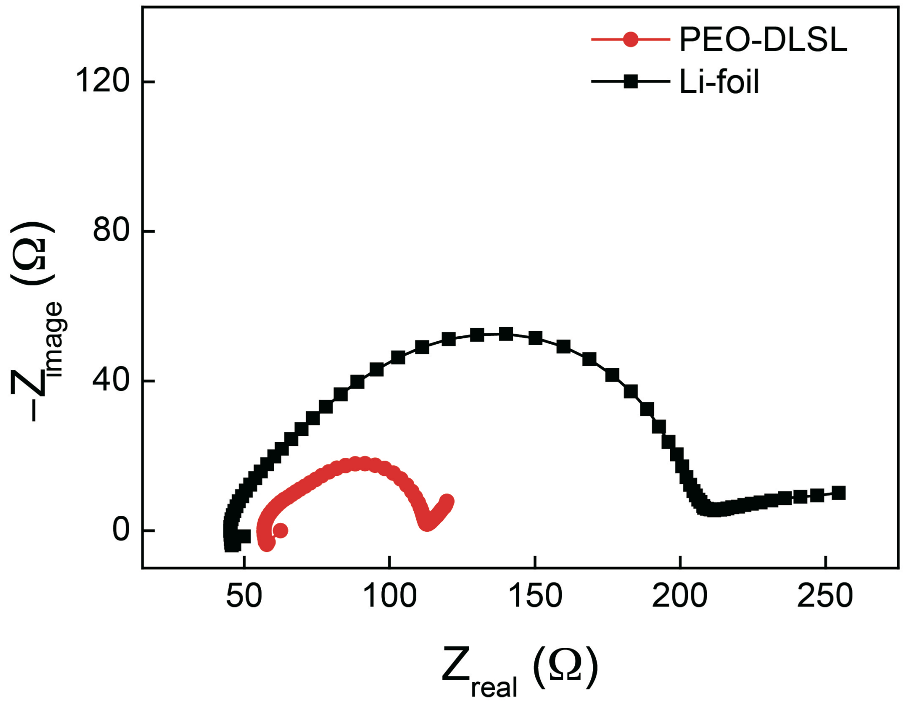

Supplementary Figure 12. Nyquist plots showing the impedance of symmetric cells using PEO-DLSL and Li foil anode.

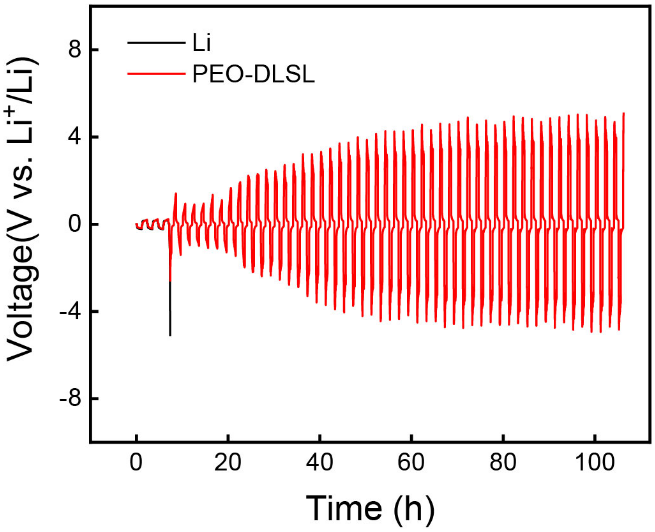

Supplementary Figure 13. Polarization voltage of Li and PEO-DLSL symmetric cells cycled at a current density of 2 mA cm^{-2}.

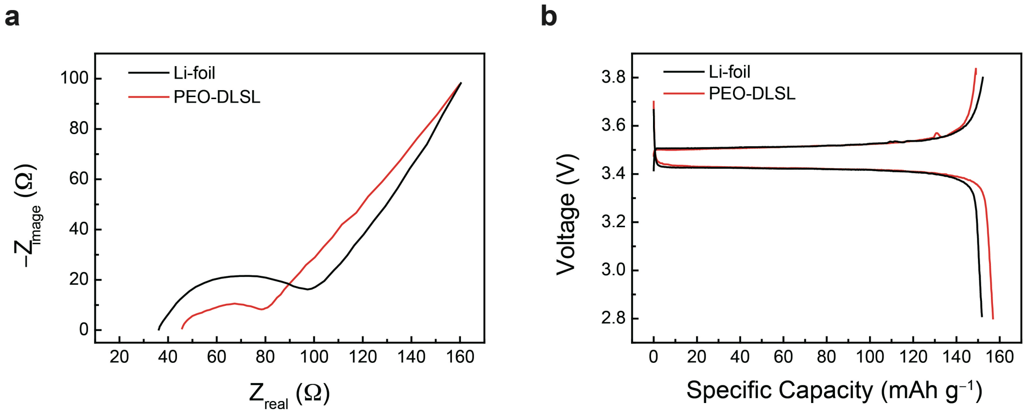

Supplementary Figure 14. The EIS results of full cells based on PEO-DLSL anode and Li foil anode. a, Nyquist plots showing the impedance of Li-LFP full cells using PEO-DLSL and Li foil anode. b, The corresponding galvanostatic charge/discharge voltage profiles of Li-LFP full cells using either PEO-DLSL or Li foil as the anode at an operating temperature of 60 °C. The current density is 0.2 C.

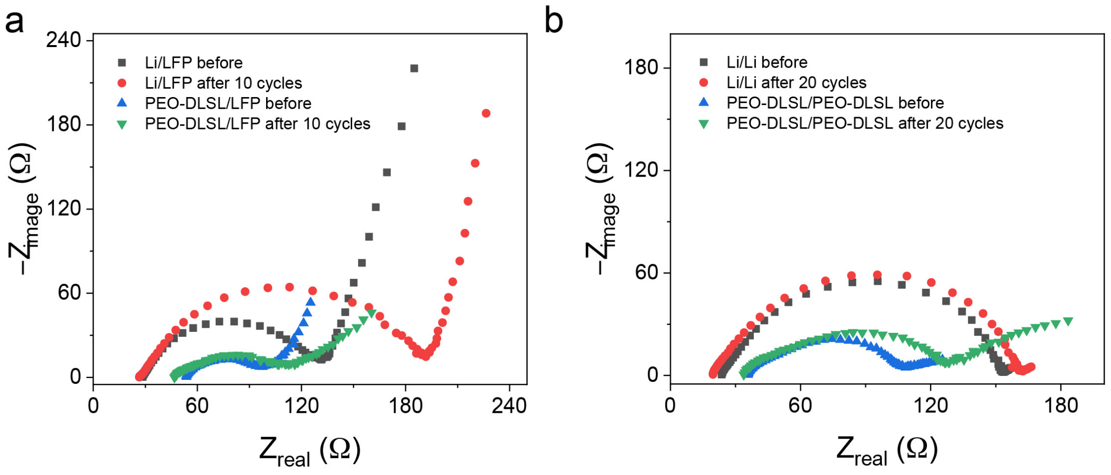

Supplementary Figure 15. The interfacial impedance variation of symmetric and full cells using PEO-DLSL and Li foil anodes before and after cycling. a, EIS curves of Li/$LiFePO_4$ and PEO-DLSL/$LiFePO_4$ full cells before and after 10 cycles. b, EIS curves of Li/Li and PEO-DLSL/PEO-DLSL symmetric cells before and after 20 cycles.

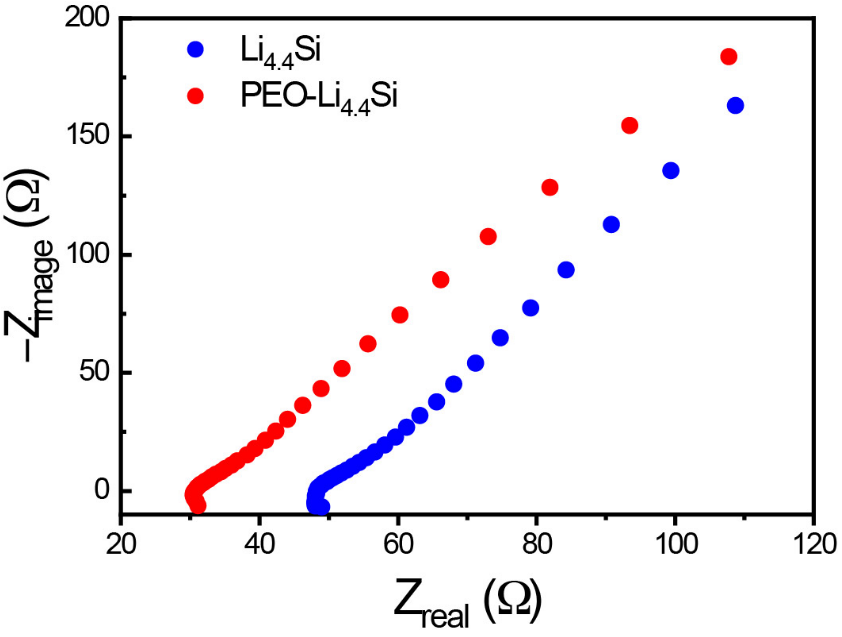

Supplementary Figure 16. Nyquist plots of $Li_{4.4}Si$ and PEO-$Li_{4.4}Si$ at 60 °C. Blue dots is corresponding to $Li_{4.4}Si$, while red circle is PEO-$Li_{4.4}Si$.

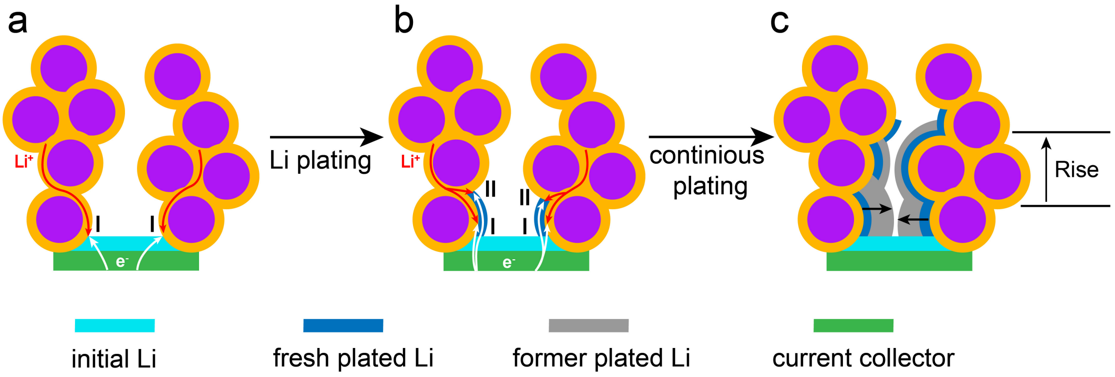

Supplementary Figure 17. Schematic illustration of Li deposition mechanism in our fabricated hierarchical composite anode. a, Li deposition at interface I. b, Generation of interface II. c, Li plating occurs at both horizontal and vertical direction to fill the whole framework.

Supplementary Table

Table 1. Parameters used in the simulation.

Symbols	Parameters	Values	References
G	shear modulus	4 GPa	1[1]
v	Poisson's ratio	0.38	1[1]
D	diffusivity	1×10^{-16} m^2 s^{-1}	2[2]
ρ	maximum concentration	3.1×10^5 mol m^{-3}	3[3]
κ	gradient energy coefficient	1.089×10^{-6} J m^{-1}	4[4]
Ω	enthalpy of mixing	0.0543 eV	
β	expansion coefficient	0.025	
k_B	Boltzmann's constant	1.38×10^{-23} J K^{-1}	
T	temperature	300 K	

Table 2. Electrochemical performance comparison of symmetric cells and solid state LFP/Li full cells based on different Li metal anode or SPE design.

Anode/SPE	symmetric cell over-potential	Cycle time (hour)	Full cell performance (areal mass loading)	Reference Number
GO-Li-PEG	125 mV-0.5 mA cm^2	900	70 mAh g^{-1}-5.0 C (~ 6.0 mg cm^{-2})	5[5]
POSS-PEG-LiTFSI	100 mV-0.5 mA cm^2	1200	90 mAh g^{-1}-2.0 C	6[6]
MOP-PEO-LiTFSI	125 mV-0.3 mA cm^2	300	85 mAh g^{-1}-2.0 C (~ 2 mg cm^{-2})	7[7]
LLZTO particles-PEO	200 mV-3.0 mA cm^2	700	75 mAh g^{-1}-2.0 C (0.9529 mg cm^{-2})	8[8]
TPU-PEO	45 mV-0.2 mA cm^2	140	75 mAh g^{-1}-2.0 C (2.1 mg cm^{-2})	9[9]
PEO in LLZTO	200 mV-0.5 mA cm^2	680	42 mAh g^{-1}-3.0 C (~ 2-3 mg cm^{-2})	10[10]
Sandwich polymer-ceramic-polymer	500 mV-0.3 mA cm^2	340	120 mAh g^{-1}-0.5 C (~ 5 mg cm^{-2})	11[11]
**PEO-DLSL**	**98 mV-0.5 mA cm^2**	**1000**	**65 mAh g^{-1}-5.0 C (2.5 mg^{-2})**	**This work**

Supplementary Note

Simulation of lithiation process and stress evolution. The computational system consists of a Si electrode and the surrounding lithium. A phase field model[12,13] is employed to simulate the lithiation process and stress evolution. The lithiation of Si electrode involves the formation of the new phase composed of $Li_{4.4}Si$, which is accompanied with a moving reaction front separating the new lithiated phase and the unlithiated phase. The lithium concentration is considered as an order parameter to characterize the phase transformation process. The evolution of the lithium concentration is governed by the Cahn-Hilliard equation[14]

$$\frac{\partial c}{\partial t} = \nabla \cdot \left(M \nabla \frac{\delta E^{tot}}{\delta c} \right), \tag{1}$$

where M is the mobility of lithium and t is time. The total energy E^{tot} consists of three parts: the chemical free energy, the interfacial energy and the elastic energy, which is written as[12,13]

$$E^{tot} = \int_V \left\{ \rho \left[\Omega c(1-c) + k_B T \left(c \ln c + (1-c) \ln (1-c) \right) \right] + \frac{\kappa}{2} |\nabla c|^2 + \frac{1}{2} \sigma_{ij} \varepsilon_{ij}^{e} \right\} dV, \tag{2}$$

where Ω is the enthalpy of mixing, which is related to the nearest-neighbor interaction strength between lithium atoms, ρ is the maximum lithium concentration accommodated in the electrode. k_B is Boltzmann's constant, T the is absolute temperature, κ is the gradient energy coefficient, σ_{ij} is the stress tensor and ε_{ij}^{e} is the elastic strain tensor. The first term in the bracket of Eq. (2) denotes the chemical free energy; the second term in Eq. (2) represents the interfacial energy; the third term incorporates the contribution of elastic energy to the system.

Based on the phase field microelasticity theory[15], we can capture the stress evolution in the electrode. The elastic equilibrium equation is solved by introducing a virtual eigenstrain $\varepsilon_{ij}^{0}(\mathbf{r})$, which is obtained by solving the following time-dependent Ginzburg-Landau kinetic equation[15]

$$\frac{\partial \varepsilon_{ij}^{0}(\mathbf{r},t)}{\partial t} = -L_{ijkl} \frac{\delta E^{\mathrm{eq}}}{\delta \varepsilon_{kl}^{0}(\mathbf{r},t)}, \tag{3}$$

where $L_{ijkl}=L_0\delta_{ij}\delta_{kl}$ is the relaxation coefficient, δ_{ij} is the Kronecker delta function, $\delta_{ij}=1$ when $i=j$ and $\delta_{ij}=0$ otherwise. In this paper, the summation convention is adopted for repeated indices. E^{eq} is the elastic energy of the equivalent system[12,13,15],

$$\begin{aligned} E^{\mathrm{eq}} &= \frac{1}{2}\int_V \left(C^0_{ijpq}\Delta S_{pqmn}C^0_{mnkl} - C^0_{ijkl}\right)C^0_{ijkl}\left[\varepsilon^0_{ij}(\mathbf{r}) - \varepsilon^c_{ij}(\mathbf{r})\right]\left[\varepsilon^0_{kl}(\mathbf{r}) - \varepsilon^c_{kl}(\mathbf{r})\right]dr^3 \\ &+ \frac{1}{2}\int_V C^0_{ijkl}\varepsilon^0_{ij}(\mathbf{r})\varepsilon^0_{kl}(\mathbf{r})d^3r - \bar{\varepsilon}_{ij}\int_V C^0_{ijkl}\varepsilon^0_{kl}(\mathbf{r})d^3r + \frac{V}{2}C^0_{ijkl}\bar{\varepsilon}_{ij}\bar{\varepsilon}_{kl} \\ &- \frac{1}{2}\int_{|\mathbf{k}|\neq 0} n_i\tilde{\sigma}^0_{ij}(\mathbf{k})\Omega_{jk}(\mathbf{n})\tilde{\sigma}^0_{kl}(\mathbf{k})' n_l \frac{d^3k}{(2\pi)^3}, \end{aligned} \tag{4}$$

where V is the total volume of the system, C^0_{ijkl} is the elastic modulus of SiO electrode and $\Delta S_{ijkl} = \left[C^0_{ijkl} - C_{ijkl}(\mathbf{r})\right]^{-1}$, $\bar{\varepsilon}_{ij} = \int_V \varepsilon_{ij}(\mathbf{r})dr^3 / V$ is the averaged strain. $\mathbf{k}$ is the wave vector in the Fourier space and $\mathbf{n} = \mathbf{k}/k$ is its unit directional vector, $\tilde{\sigma}^0_{ij}(\mathbf{k})$ is the Fourier transform of $\sigma^0_{ij}(\mathbf{r})$ and $\tilde{\sigma}^0_{kl}(\mathbf{k})'$ is the complex conjugate of $\tilde{\sigma}^0_{ij}(\mathbf{k})$, $\Omega_{ij}(\mathbf{n})$ is Green function tensor defined as the inverse tensor of $C^0_{ijkl}n_k n_l$. $\varepsilon^c_{ij}(\mathbf{r})$ is the chemical eigenstrain and it is defined as $\varepsilon^c_{ij}(\mathbf{r}) = \beta\left(c(\mathbf{r}) - c_0\right)\delta_{ij}$, where β is the expansion coefficient and c_0 is the reference concentration. Once $\varepsilon^0_{ij}(\mathbf{r},t)$ is determined, the elastic strain is given by[15]

$$\varepsilon^e_{ij}(\mathbf{r}) = \bar{\varepsilon}_{ij} + \frac{1}{2}\int_{|\mathbf{k}|\neq 0} \left(n_i\Omega_{jk} + n_j\Omega_{ik}\right)\tilde{\sigma}^0_{kl}(\mathbf{k})n_l\, e^{i\mathbf{k}\cdot\mathbf{r}}d^3\mathrm{k}/(2\pi)^3 - \varepsilon^c_{ij}, \tag{5}$$

The stress and the elastic strain obeys Hooke's law[15]

$$\sigma_{ij}(\mathbf{r}) = C_{ijkl}(\mathbf{r})\varepsilon^e_{kl}(\mathbf{r}) \tag{6}$$

It should be pointed out that the silicon electrode suffers from large deformation due to accommodating large amount of lithium. Here, the phase field model based on the framework of small deformation is used to simulate the tendency of the stress evolution. For the convince of numerical simulation, the following dimensionless parameters are introduced

$$t^* = \frac{Dt}{l^2} \qquad \kappa^* = \frac{\kappa}{c_m k_B T l^2}$$
$$\sigma_{ij}^* = \frac{\sigma_{ij}}{G} \qquad \Omega^* = \frac{\Omega}{k_B T} \quad \nabla^* = \nabla l \tag{7}$$

where D is the diffusivity of lithium, which is related to the mobility by the Einstein relation $D = k_B T M$. The characteristic length l is set as 1 nm. The parameters of silicon electrode used in our simulation are summarized in Supplementary Table 1.

Supplementary References

1. Cui, Z., Gao, F., Cui, Z. & Qu, J. A second nearest-neighbor embedded atom method interatomic potential for Li-Si alloys. *J. Power Sources* **207**, 150–159, (2012).
2. Liu, X. H. *et al.* Anisotropic Swelling and Fracture of Silicon Nanowires during Lithiation. *Nano Lett.* **11**, 3312–3318, (2011).
3. Xu, Y. H., Yin, G. P. & Zuo, P. J. Geometric and electronic studies of $Li_{15}Si_4$ for silicon anode. *Electrochim. Acta* **54**, 341–345, (2008).
4. Chen, L. *et al.* A Phase-Field Model Coupled with Large Elasto-Plastic Deformation: Application to Lithiated Silicon Electrodes. *J. Electrochem. Soc.* **161**, F3164–F3172, (2014).
5. Liu, Y. *et al.* Transforming from planar to three-dimensional lithium with flowable interphase for solid lithium metal batteries. *Sci. Adv.* **3**, eaao0713, (2017).
6. Pan, Q., Smith, D. M., Qi, H., Wang, S. & Li, C. Y. Hybrid Electrolytes with Controlled Network Structures for Lithium Metal Batteries. *Adv. Mater.* **27**, 5995–6001, (2015).
7. Zhou, W., Gao, H. & Goodenough, J. B. Low-Cost Hollow Mesoporous Polymer Spheres and All-Solid-State Lithium, Sodium Batteries. *Adv. Energy Mater.* **6**, 1501802, (2016).
8. Zhang, J. *et al.* Flexible and ion-conducting membrane electrolytes for solid-state lithium batteries: Dispersion of garnet nanoparticles in insulating polyethylene oxide. *Nano Energy* **28**, 447–454, (2016).
9. Tao, C. *et al.* A promising TPU/PEO blend polymer electrolyte for all-solid-state lithium ion batteries. *Electrochim. Acta* **257**, 31–39, (2017).
10. Chen, L. *et al.* PEO/garnet composite electrolytes for solid-state lithium batteries: From "ceramic-in-polymer" to "polymer-in-ceramic". *Nano Energy* **46**, 176–184, (2018).
11. Zhou, W. *et al.* Plating a Dendrite-Free Lithium Anode with a Polymer/Ceramic/Polymer Sandwich Electrolyte. *J. Am. Chem. Soc.* **138**, 9385–9388, (2016).
12. Chang, L., Lu, Y., He, L. & Ni, Y. Phase field model for two-phase lithiation in an arbitrarily shaped elastoplastic electrode particle under galvanostatic and potentiostatic operations. *Int. J. Solids Struct.* **143**, 73–83, (2018).
13. Lu, Y. & Ni, Y. Effects of particle shape and concurrent plasticity on stress generation

during lithiation in particulate Li-ion battery electrodes. *Mech. Mater.* **91**, 372–381, (2015).

14. Cahn, J. W. & Hilliard, J. E. Free Energy of a Nonuniform System. I. Interfacial Free Energy. *J. Chem. Phys.* **28**, 258–267, (1958).

15. Wang, Y. U., Jin, Y. M. & Khachaturyan, A. G. Phase field microelasticity theory and modeling of elastically and structurally inhomogeneous solid. *J. Appl. Phys.* **92**, 1351–1360, (2002).
