# Supplementary material for: Diatomite derived hierarchical hybrid anode for high performance all-solid-state lithium metal batteries
Source: Nat Commun. 2019 Jun 6;10:2482. doi: 10.1038/s41467-019-10473-w (PMC6554300; doi:10.1038/s41467-019-10473-w)
Supplement: Supplementary file 3 — Description of Additional Supplementary Files [file 41467_2019_10473_MOESM3_ESM.pdf]

Description of Additional Supplementary Files

File Name: Supplementary Movie-1
Description: The three-dimensional (3D) structures of pristine DF characterized by the soft X-ray tomography;

File Name: Supplementary Movie-2
Description: The three-dimensional (3D) structures of pristine DF-SiO characterized by the soft X-ray tomography;

File Name: Supplementary Movie-3
Description: The three-dimensional (3D) structures of pristine DF-Si characterized by the soft X-ray tomography.
